# *Quercus acuta* Acorn Bran Extract Enhances Wound Healing by Promoting Human Dermal Fibroblast Migration and Antioxidant Activity

**DOI:** 10.3390/ph19030481

**Published:** 2026-03-15

**Authors:** So-An Lim, Tae Hyun Son, Hye-Lim Shin, Dongsoo Kim, Jun-Hyuck Yoon, Hwan-Gyu Kim, Hyunmo Choi, Shin-Hye Kim, Sik-Won Choi

**Affiliations:** 1 Pharmacogenomics Research Center, Inje University College of Medicine, Busan 47392, Republic of Korea; limsa0223@naver.com; 2 Forest Biomaterials Research Center, National Institute of Forest Science (NIFoS), Jinju 52817, Republic of Korea; snoopyegg@korea.kr (T.H.S.); hlims0901@korea.kr (H.-L.S.); skimds@korea.kr (D.K.); jhyoon7988@korea.kr (J.-H.Y.); 3 Department of Biological Sciences, Jeonbuk National University, Jeonju 54896, Republic of Korea; hgkim@jbnu.ac.kr; 4 Department of Forest Bioresources, National Institute of Forest Science (NIFoS), Suwon 16631, Republic of Korea; choihyunmo@korea.kr; 5 Forest Medicinal Resources Research Center, National Institute of Forest Science (NIFoS), Yeongju 36040, Republic of Korea

**Keywords:** *Quercus acuta*, evergreen oaks, wound healing, skin regeneration, tissue regeneration

## Abstract

**Background/Objectives**: Wound repair-associated processes and the antioxidant properties of natural products play critical roles in skin wound healing and barrier restoration. Wound healing is a complex process characterized by a series of interconnected events that facilitate the self-repair of the skin following injury. **Methods**: This study aimed to evaluate the effects of *Quercus acuta* acorn bran extract (QAABE) on wound healing using human dermal fibroblast (HDF) cell cultures treated with QAABE. Additionally, in vivo experiments were conducted using a mouse model of skin injury to assess the wound-healing potential of the extract. **Results**: The results indicated that QAABE enhanced wound healing in vitro by upregulating extracellular matrix-related markers, including vimentin, Col1a1, Col3a1, endothelin, fibronectin, and VEGF at the mRNA level, and increasing the protein expression of vimentin, COL1A1, endothelin, and α-SMA. QAABE also exhibited reactive oxygen species (ROS)-scavenging activity. In the mouse skin injury model, QAABE treatment accelerated wound closure and was associated with reduced inflammatory responses. **Conclusions**: These findings suggest that QAABE may promote wound-healing-related responses in both in vitro and in vivo models, supporting its potential as a candidate for further investigation in wound-healing research.

## 1. Introduction

Natural products have demonstrated their effectiveness in promoting wound healing and reestablishing the skin’s barrier function, largely because of their anti-inflammatory, antioxidant, and antimicrobial characteristics [1]. These beneficial effects are largely attributed to phytochemicals, including alkaloids, essential oils, flavonoids, tannins, saponins, and phenolic compounds [2]. The process of wound healing takes place in three distinct stages: inflammation, proliferation, and tissue remodeling [3]. Although the inflammatory stage is crucial for initiating the healing process, excessive inflammation can hinder recovery, potentially resulting in fibrosis, scarring, and delayed healing [4,5]. During this stage, various cellular mediators such as cytokines and nitric oxide (NO) are produced. Therefore, regulating inflammation is essential for achieving optimal wound-healing outcomes.

Reactive oxygen species (ROS) are oxygen-derived molecules that serve as oxidants and can contribute to cellular impairment. Paradoxically, they also fulfill a critical function in facilitating typical wound-healing responses [6]. At moderate levels, ROS play a crucial role in protecting tissues against infections and facilitate wound healing by activating pathways that promote cell survival [7,8]. However, excessive ROS generation can lead to cytotoxicity and exacerbate inflammation, inhibiting the healing process [9]. Antioxidants, particularly polyphenols, donate electrons to ROS, thereby preventing the oxidation of essential biological molecules such as proteins and DNA. Moreover, polyphenols trigger complex reaction cascades that transform ROS into more stable forms, helping to sustain safe concentrations of ROS in wounded sites and facilitating the recovery mechanism [10,11].

The proliferation stage is characterized by angiogenesis, which refers to the generation of novel blood vessels, as well as collagen accumulation and granulation tissue development. Fibroblasts serve a crucial role during this period by producing components of the extracellular matrix and promoting tissue regeneration [12]. The final stage, tissue remodeling, encompasses the ripening of newly formed tissue and the reorganization of collagen fibers, which is crucial for restoring the structural stability and functionality of the skin [13].

*Quercus acuta* Thunb. is extensively cultivated for both nutritional and decorative purposes in East Asia [14]. In Korean cuisine, jelly prepared from the acorns of *Q. acuta* Thunb. is particularly popular [14]. Previous studies have indicated that these acorns exhibit inhibitory effects against UVB-induced photoaging and can reduce serum uric acid levels through xanthine oxidase inhibition [14,15]. Additionally, various extracts derived from the leaves of *Q. acuta* Thunb. have demonstrated antioxidant and antibacterial properties [15]. Despite these established benefits, the potential wound-healing effects of *Q. acuta* Thunb. remain understudied. To address this gap in the literature, this research aimed to study the prospective role of *Q. acuta* acorn bran extract (QAABE) in facilitating skin wound healing.

## 2. Results

### 2.1. Q. acuta Acorn Bran Extract (QAABE) Enhances Migration Activity in HDF Cells

**Figure 1:**
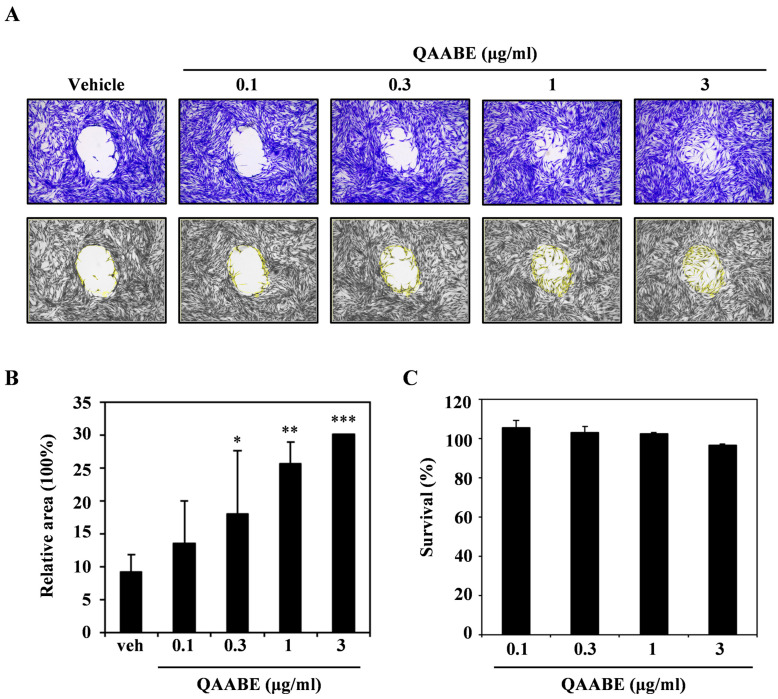
*Q. acuta* acorn bran extract (QAABE) promotes wound healing in HDF cells. (**A**) HDF cells were incubated in a migration plate for 24 h, followed by treatment with QAABE. The cells were then cultured for an additional 24 h, fixed, and stained using a cell staining solution. The bottom panel shows grayscale images of the cells, with the wound closure area highlighted in yellow. (**B**) The relative wound area was measured using ImageJ (ver. 1.54g). * *p* < 0.05, ** *p* < 0.01, *** *p* < 0.001 (compared to the vehicle control). (**C**) The effect of QAABE on the viability of HDF cells was assessed using the CCK-8 assay. Data are presented as the mean ± SD (*n* = 3).

To evaluate the migration effects of QAABE in vitro, a wound-healing assay was performed using HDF cells. As shown in Figure 1A, QAABE treatment promoted a dose-dependent closure of the wound area. Statistical analysis of these results confirmed that QAABE significantly enhanced wound repair in a concentration-dependent manner (Figure 1B). This finding is significant, as it demonstrates cell migration independent of cell viability (Figure 1C). These findings indicate that QAABE possesses wound-healing properties in HDF cells.

### 2.2. QAABE Upregulates Migration-Mediated Molecules in HDF Cells

**Figure 2:**
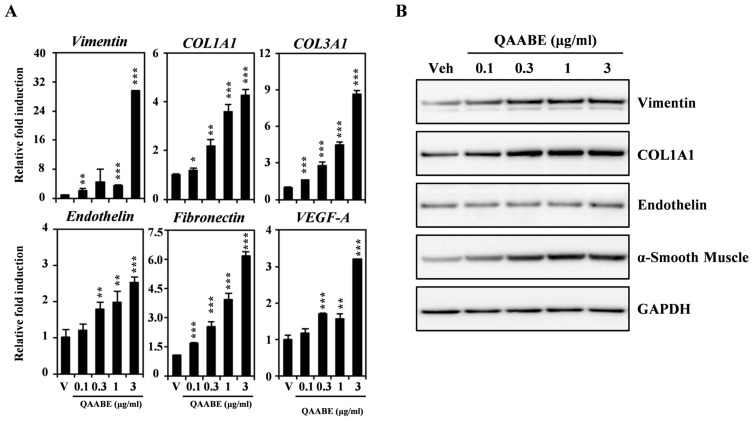
QAABE enhances the expression of molecular markers associated with wound healing in HDF cells. (**A**) The mRNA expression levels in HDF cells treated with either V or QAABE (0.1, 0.3, 1, and 3 μg/mL) were measured using qRT-PCR. V indicates the vehicle control. *Actin* and *HPRT1* served as internal controls; * *p* < 0.05, ** *p* < 0.01, *** *p* < 0.001 (compared to the vehicle control). Data are presented as the mean ± SD (*n* = 3). (**B**) The effect of QAABE on the protein expression levels of wound-healing markers was assessed using Western blot analysis, with GAPDH as the internal control. The results are representative of three independent experiments that yielded consistent findings.

To elucidate the wound healing effects of QAABE, we investigated the expression of molecular markers associated with wound healing in HDF cells. As shown in Figure 2A, QAABE treatment resulted in a dose-dependent increase in the mRNA expression levels of various molecular markers, including *vimentin*, *Col1a1*, *Col3a1*, *endothelin*, *fibronectin*, and *vascular endothelial growth factor (VEGF)*. Additionally, the protein expression levels of molecular markers pertinent to wound healing, including vimentin, COL1A1, endothelin, and α-SMA, were upregulated following QAABE treatment (Figure 2B). Collectively, these findings suggest that QAABE enhances cell migration by upregulating the expression of molecular markers associated with the wound-healing process.

### 2.3. QAABE Mitigates ROS Induced by H_2_O_2_ in HDF Cells

**Figure 3:**
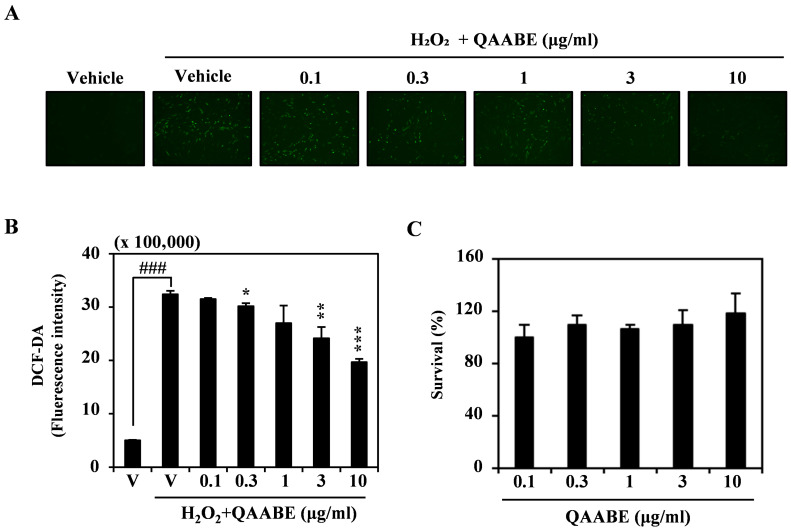
QAABE reduces levels of ROS in HDF cells. (**A**) HDF cells were starved for 3 h and pretreated with 10 μM of 2,7-dichlorofluorescein diacetate (DCF-DA) and QAABE for 1 h. Subsequently, cell morphology was observed using a fluorescence microscope. (**B**) DCF-DA fluorescence intensity was measured using a fluorescence spectrophotometer at wavelengths of 475–535 nm. V indicates the vehicle control. ### *p* < 0.001 (versus vehicle control); * *p* < 0.05; ** *p* < 0.01; *** *p* < 0.001 (compared to the H_2_O_2_-treated group). (**C**) The effect of QAABE on HDF cell viability was evaluated using the CCK-8 assay. Data are expressed as the mean ± SD (*n* = 3).

To investigate the antioxidant effects of QAABE, we examined its ability to reduce ROS levels induced by H_2_O_2_ in HDF cells. QAABE treatment resulted in a dose-dependent inhibition of intracellular ROS levels in HDF cells (Figure 3A). ROS levels were quantified using DCF-DA. The results further confirmed that QAABE treatment resulted in a concentration-dependent decrease in ROS levels (Figure 3B). Notably, these reductions in ROS levels were not associated with changes in cell viability at the tested concentrations (Figure 3C).

### 2.4. QAABE Ameliorates H_2_O_2_-Induced Impairment of Wound Healing in HDF Cells

**Figure 4:**
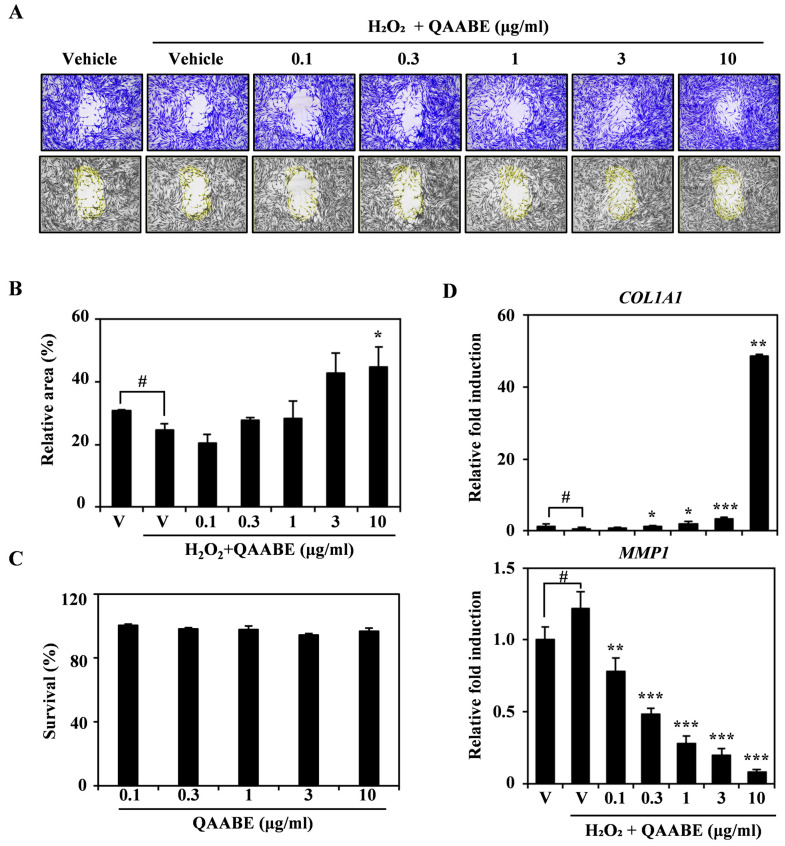
Effects of QAABE on H_2_O_2_-induced wound healing in HDF cells. (**A**) HDF cells were cultured in a migration plate with either a vehicle control or various concentrations of QAABE (0.1, 0.3, 1, 3, and 10 μg/mL) in the presence of H_2_O_2_ (8.8 mM). Following the wound-healing process, cells were fixed and stained. (**B**) The wound area was quantified using ImageJ. V indicates the vehicle control. Statistical significance was determined as # *p* < 0.05 (compared to the vehicle control) and * *p* < 0.05 (compared to the H_2_O_2_-treated group). (**C**) The cytotoxicity of QAABE was assessed using the CCK-8 assay. (**D**) mRNA expression levels in HDF cells treated with either V or QAABE (0.1, 0.3, 1, 3, and 10 μg/mL) in the presence of H_2_O_2_ (500 μM) were measured using qRT-PCR. V indicates the vehicle control. Actin served as the internal control, and reactions were performed in triplicate. Statistical significance was indicated as # *p* < 0.05 (compared to the vehicle control); * *p* < 0.05; ** *p* < 0.01; *** *p* < 0.001 (compared to the H_2_O_2_-treated group). Data are presented as the mean ± SD (*n* = 3).

QAABE treatment enhanced cell migration, which had been inhibited by H_2_O_2_ in HDF cells (Figure 4A). Similarly to the results presented in Figure 4A, analysis of the wound closure area indicated that QAABE treatment facilitated the wound recovery in a dose-dependent manner (Figure 4B). This effect was independent of cell viability, as determined by the CCK-8 assay (Figure 4C). Furthermore, QAABE treatment significantly increased Col1a1 mRNA expression, which had been downregulated by H_2_O_2_, in a dose-dependent manner. Conversely, QAABE treatment suppressed the mRNA expression of Matrix metalloproteinase-1 (MMP1), which had been elevated by H_2_O_2_ (Figure 4D).

### 2.5. QAABE Facilitates Wound Recovery In Vivo in Mouse Injury Model

**Figure 5:**
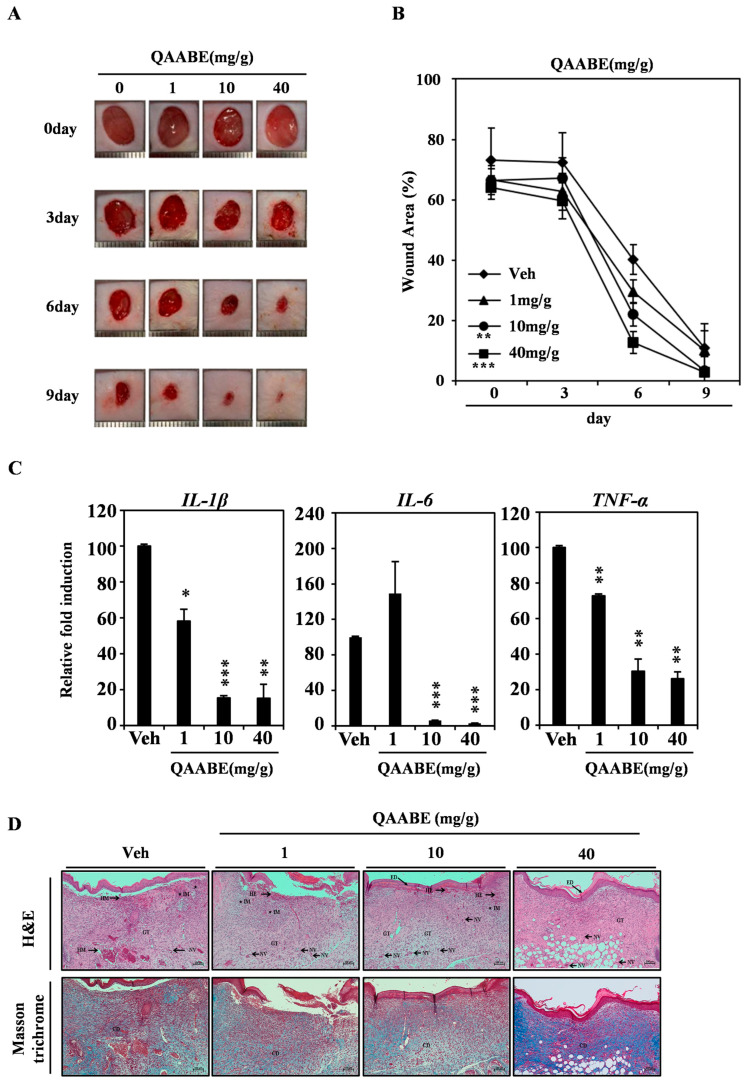
QAABE promotes wound healing in an in vivo mouse injury model. (**A**) A full-thickness skin excision measuring 8 mm in diameter was created on the back of each mouse. QAABE was applied to the wounded area daily for 9 d. (**B**) The area was measured using a digital caliper. ** *p* < 0.01; *** *p* < 0.001 (compared to the vehicle control at 9 d). Data are expressed as the mean ± SD (*n* = 5). (**C**) The mRNA expression levels were analyzed using qRT-PCR. * *p* < 0.05; ** *p* < 0.01; *** *p* < 0.001 (compared to the vehicle control). Data are expressed as the mean ± SD (*n* = 5). (**D**) Skin tissue samples were fixed in 3.7% formalin for 24 h, sectioned to a thickness of 4 μm, and analyzed using hematoxylin and eosin (H&E) staining and Masson’s trichrome staining. Arrows indicate neovascularization within the granulation tissue, and ★ symbols indicate inflammatory cell infiltration. GT: granulation tissue formation, HE: hemorrhage, IM: inflammation, NV: neovascularization, ED: epithelialization, CD: collagen deposition. Bar = 100 μm.

To estimate the wound-healing effects of QAABE in vivo, we conducted an animal experiment using a mouse skin injury model. QAABE treatment significantly accelerated wound healing in the model in a dose-dependent manner (Figure 5A). The percentage of wound area reduction was greater in the 40 mg/g QAABE-treated group compared to the vehicle-treated group (Figure 5B). Additionally, QAABE treatment led to a dose-dependent reduction in the mRNA levels of inflammatory cytokines, including IL-1β, IL-6, and TNF-α, at the wound site (Figure 5C). H&E staining revealed that key indicators associated with wound healing were enhanced by QAABE treatment in a dose-dependent manner. Skin condition scores also improved in a dose-dependent manner (Table 1). Additionally, Masson’s trichrome staining demonstrated an increase in collagen deposition, indicated by a shift to blue coloration, following QAABE treatment (Figure 5D). Collectively, these findings suggest that QAABE exhibits significant wound-healing and anti-inflammatory properties in vivo.

### 2.6. QAABE Contains a Diverse Array of Polyphenolic Compounds

Among the polyphenolic compounds analyzed, protocatechuic acid, catechin, gallic acid, epicatechin, and ellagic acid were present in the highest concentrations in QAABE. This assessment was conducted through a comparison of 12 standard compounds using LC-MS/MS at a concentration of 100 ppb (Table 2; Supplementary Figure S1).

## 3. Discussion

This study aimed to elucidate the effects of QAABE on skin injury. The genus Quercus is categorized into deciduous and evergreen oaks, both of which are recognized for their acorn [16,17]. Historically, acorns have been used in the treatment of burns owing to their wound-healing properties [18]. Quercus glauca has been shown to effectively facilitate skin wound healing both at the cellular level and in vivo [19]. Building upon these findings, the present study extends the investigation to QAABE, highlighting species-specific differences in phenolic composition and antioxidant capacity within the genus. Consequently, we conducted experiments using the bran of acorns from *Q. acuta*, an evergreen oak species. The significance of this study lies in its focus on the acorn seed coats, which are typically discarded as waste.

We demonstrated that the healing of wounded HDF cells occurred more rapidly with the application of QAABE compared to the vehicle control. Notably, this effect was not associated with cell viability or cytotoxicity. Vimentin plays a crucial role in the wound-healing process, supporting fibroblast function during both the proliferation and remodeling phases, particularly in conjunction with Transforming growth factor (TGF) signaling [20]. Collagen types Col1a1 and Col3a1 contribute to wound healing by attracting fibroblasts and stimulating new collagen synthesis in the affected area. Consequently, enhancing collagen expression is essential for effective wound healing [21]. Endothelin (ET-1) has traditionally been identified as a hormone that regulates blood pressure through vasoconstriction [22]. However, it also facilitates wound healing by enhancing the contractile activity of fibroblasts, which is vital for wound closure and dermal reconstitution [23,24]. Fibronectin serves multiple functions in the wound-healing process, primarily by promoting cellular adhesion and mediating cell growth and migration [25]. VEGF promotes wound recovery through various mechanisms, including collagen deposition, angiogenesis, and epithelial tissue expansion [26]. The expression of α-SMA is indicative of fully differentiated myofibroblasts [23]. Collectively, these findings suggest that QAABE treatment enhanced the expression of biomarkers associated with wound healing in a dose-dependent manner.

Elevated levels of ROS have been shown to impede wound closure, and targeting oxidative stress can accelerate clinical wound healing [24]. Our findings indicate that QAABE inhibited intracellular ROS induced by H_2_O_2_ in HDF cells, without affecting cell viability or cytotoxicity, suggesting an antioxidant function of QAABE. We used an H_2_O_2_-induced wound model to simulate delayed wound healing due to inflammation, and our results demonstrated that QAABE exhibited a wound-healing effect in HDF cells. Prolonged increases in MMP1 can extend the inflammatory phase of wound healing, leading to chronic wound conditions [27]. Notably, both MMP1, which is associated with inflammation, and Col1a1, which is linked to wound healing, were regulated by QAABE treatment.

QAABE significantly reduced intracellular ROS levels compared to the H_2_O_2_-treated control, suggesting a potential antioxidative effect. Excessive ROS is known to impair fibroblast function and delay wound healing [24]. The observed ROS-scavenging activity may therefore contribute to enhanced extracellular matrix regulation and accelerated wound closure in vivo. These findings further support species-specific differences in phenolic composition within the *Quercus* genus, which may influence the magnitude of antioxidant-mediated wound-healing effects.

Subsequently, we employed a mouse skin injury model to elucidate the in vivo effects of QAABE on wound healing. Mice are considered an optimal preclinical model due to their cost-effectiveness and susceptibility to genetic modifications, which facilitate mechanistic investigations [28]. Plant extracts have been shown to enhance the healing process by promoting growth factors, proliferation, development, and motility of specific cells involved in wound healing [29]. In our study, QAABE accelerated wound recovery in a concentration-dependent manner in the mouse wound model. To ensure experimental precision, we followed to established protocols for the murine wound model [30]. According to existing literature, gene expression studies on wound samples collected at various time points post-injury can provide insights into gene expression alterations throughout the stages of wound healing [30]. However, in this study, we conducted qRT-PCR analysis on Day 9, as we aimed to compare the vehicle group with the extract-treated group. QAABE treatment attenuated inflammatory gene expression and improved indicators and scores related to wound healing, as evidenced by H&E staining and Masson’s trichrome staining.

QAABE contains various polyphenolic compounds and protocatechuic acid-rich *Trianthema portulacastrum* leaves have been shown to promote dermal wound healing [31]. Additionally, a previous study demonstrated that a bilayer film composed of catechin and gelatin exhibited favorable wound-healing properties [32]. It is therefore plausible that QAABE exerts its wound-healing activity through the presence of protocatechuic acid and catechin. Collectively, QAABE may represent a promising natural resource for further investigation in wound-healing research.

## 4. Materials and Methods

### 4.1. Q. acuta Acorn Bran (QAAB) Preparation

On 13 November 2020, acorns from the *Q. acuta* species were collected in Jinju, Gyeongsangnam-do, Republic of Korea. Researcher Hyun-Jun Kim at the Forest Medicinal Resources Research Center classified the acorns, labeled FMRC-201113A1-3. The acorns were rinsed with sterile water and air-dried at 50 °C for 3 d to remove moisture. The bran was then separated from the seeds, preparing them for further analysis.

### 4.2. QAAB Extraction

The desiccated QAAB was extracted using DDW in an autoclave at 121 °C for 15 min. The extract was centrifuged at 1450× *g* for 5 min and filtered through a 40-μm mesh. It was then vacuum evaporated and freeze-dried into a fine powder. Finally, the freeze-dried QAABE was dissolved in DMSO to a stock concentration of 30 mg/mL. The solution was clear and remained stable without visible precipitation under experimental conditions. The final DMSO concentration in all cell-based assays was ≤0.1%, and equivalent DMSO concentrations were used in vehicle controls.

### 4.3. Reagents and Antibodies

All experiments proceeded as planned, with minor adjustments [19]. We obtained FBS and antibiotics from Gibco (Thermo Fisher Scientific, Waltham, MA, USA), TRIzol from Invitrogen (Carlsbad, CA, USA), and DMSO from Sigma-Aldrich (St. Louis, MO, USA). For oxidative experiments, we acquired H_2_O_2_ from Duksan Science (Seoul, Republic of Korea). Our antibody arsenal included β-actin (sc-47778) and several HRP-conjugated secondary antibodies from Santa Cruz Biotechnology (Santa Cruz, CA, USA). We also included antibodies against COL1A1 (#72026), α-smooth muscle actin (α-SMA) (#19245), and vimentin (#5741) from Cell Signaling Technology (Danvers, MA, USA), as well as the antibody for endothelin-1 (ET-1, PA3-067) from Invitrogen.

### 4.4. Cell Culture

HDFs were sourced from Cefo Bio (Seoul, Republic of Korea) and thawed in a 37 °C water bath, with a passage number of one. They were cultured in DMEM with 10% FBS and antibiotics, incubated at 37 °C in a 5% CO_2_ environment using a Panasonic incubator (model MCO-170AIC-PK, Tokyo, Japan). The culture medium was refreshed every 2 to 3 d.

### 4.5. Migration Assay

The Radius™ 96-Well Cell Migration Assay Kit (Cell Biolabs, Inc., San Diego, CA, USA) was used to assess the wound-healing properties of the compounds. Each well received 100 μL of Radius™ Gel Pretreatment Solution. HDFs were washed, seeded at 1 × 10^4^ cells per well, and incubated for 24 h. After washing with fresh medium, 100 μL of 1× Radius™ Gel Removal Solution was added, followed by a 30 min incubation. The cells were cultured for another 24 h in serum-free medium with QAABE at concentrations of 0.1, 0.3, 1, and 3 µg/mL, alongside a positive control of 2% FBS. After incubation, the medium was removed, and the cells were fixed in 10% formalin for 5 min, rinsed with DDW, and stained. The recovery area was quantified using ImageJ version 1.8 (NIH, Bethesda, MD, USA).

### 4.6. Cell Cytotoxicity Assay

A Cell Counting Kit-8 (CCK-8) assay was conducted to evaluate the effects of QAABE on the viability of HDFs. Cells were seeded in 96-well plates and incubated for 24 h with the indicated concentrations of QAABE. The concentration range (0.1–10 µg/mL) was selected based on the cytotoxicity results, which confirmed the absence of significant toxicity within this range. After incubation, CCK-8 reagent was added to each well, and the plates were incubated for 30 min. The optical density was then measured at 450 nm using a spectrophotometer.

### 4.7. RNA Isolation and Quantitative Reverse Transcription Polymerase Chain Reaction (qRT-PCR)

HDFs were plated at a density of 1 × 10^5^ cells/mL in a 6-well plate and incubated overnight. After incubation, the cells were treated with QAABE at specified concentrations. To evaluate the expression of inflammatory genes, cells were treated with 500 µM H_2_O_2_ for 24 h. This lower concentration was selected to assess cytotoxicity and gene expression while minimizing excessive cell death. Following the experimental procedures, total RNA was extracted from both HDFs and mouse skin tissues using TRIzol reagent, following the manufacturer’s guidelines. complementary DNA (cDNA) was synthesized from 1 μg of total RNA with the RevertAid First Strand cDNA Synthesis Kit (Thermo Fisher Scientific), adhering to the manufacturer’s instructions for qRT-PCR. Primer pairs were designed using the online Primer3 software (v. 0.4.0) [33] (Table 3). SYBR Green-based qRT-PCR was performed using the QuantStudio™ 5 real-time PCR System (Thermo Fisher Scientific) with PowerUp™ SYBR™ Green Master Mix (Thermo Fisher Scientific). All sample mixtures were prepared in triplicate, and the data were analyzed using the 2^−ΔΔCT^ method as described by Livak and Schmittgen [34]. *Hypoxanthine phosphoribosyl-transferase 1 (HPRT1)* or *β-actin (ACTB)* served as the internal control.

### 4.8. Western Blotting

Following treatment with QAABE or a vehicle control, cells were lysed on ice for 10 min using radioimmunoprecipitation assay lysis buffer (Cell Signaling Technology) contained protease inhibitors. Proteins were extracted from the supernatant by centrifugation at 15,000× *g* for 15 min. Protein concentrations in the lysates were determined using a detergent-compatible protein assay kit (Bio-Rad, Hercules, CA, USA). Subsequently, proteins were separated by sodium dodecyl sulfate polyacrylamide gel electrophoresis and transferred to polyvinylidene difluoride membranes (Merck Millipore, Darmstadt, Germany). The membranes were incubated with the following primary antibodies: mouse monoclonal anti-β-actin at 1:500, rabbit monoclonal anti-COL1A1 at 1:1000, rabbit monoclonal anti-α-SMA at 1:1000, rabbit polyclonal anti-ET-1 at 1:1000, and rabbit monoclonal anti-vimentin at 1:1000. For several secondary antibodies, anti-mouse, anti-goat and anti-rabbit at 1:3000 were used. Clarity Western ECL Substrate (Bio-Rad) was adopted for blot development, and protein bands were imaged using the ChemiDoc XRS+ system (Bio-Rad).

### 4.9. 2,7-Dichlorofluorescein Diacetate (DCF-DA) Staining

The DCF-DA assay (Invitrogen) measured ROS levels in HDFs cultured at 1 × 10^5^ cells/mL in a 96-well black plate for 24 h. After adding serum-free medium to induce cell starvation for 3 h, oxidative stress was induced with H_2_O_2_. Following starvation, 100 μL of 10 μM DCF-DA was added, and cells were labeled with DCF-DA for 30 min. Following incubation with DCF-DA, cells were exposed to a combined treatment of 8.8 mM H_2_O_2_ and QAABE at designated concentrations for 1 h. The H_2_O_2_ concentration (8.8 mM) was selected for short-term exposure (≤2 h) to induce sufficiently detectable intracellular ROS levels for fluorescence measurement. Fluorescence-labeled cells were observed with a DMi8 inverted fluorescence microscope (Leica, Wetzlar, Germany), and fluorescence intensity was determined at 475 nm and 535 nm using a spectrophotometer (SpectraMax iD3, Molecular Devices, Sunnyvale, CA, USA).

### 4.10. Cell-Free DCFH Oxidation Assay

To evaluate potential probe-level interference of QAABE in the DCF-DA assay, a cell-free DCFH oxidation assay was performed. DCFH was incubated with H_2_O_2_ in DPBS in the absence of cells, and fluorescence intensity was measured in the presence of increasing concentrations of QAABE. Vehicle controls contained DCFH + H_2_O_2_ + DMSO. In addition, QAABE-only wells (DPBS + QAABE) were included to evaluate extract-derived background fluorescence. Fluorescence intensity was measured using a fluorescence spectrophotometer at excitation and emission wavelengths of 475 and 535 nm, respectively.

### 4.11. Ethical Considerations in Animal Testing

This research followed the Standard Protocol for Animal Studies set by the Department of Laboratory Animal Resources at Yonsei Hospital Biomedical Research Institute, with approval from the Institutional Animal Care and Use Committee (Permit No. 2021-0184). Animal experiments were designed to use the minimum number of animals necessary while ensuring minimal discomfort.

### 4.12. Arrive Guidelines

A pre-clinical study was designed as a prospective, randomized, and blinded trial to assess the wound healing effects of QAABE. The sample size for each group was determined using G-power software (version 3.1.9.7) based on preliminary studies. Based on the power calculation, five mice were allocated per treatment group. No mortality was observed during the experimental period. The allocation of animals to treatment groups was conducted using a computer-generated randomization tool (https://www.randomizer.org/, accessed on 1 December 2021). Each animal was assigned a unique identification number, and cages were numbered according to their position on the rack. The following parameters were evaluated: digital photography, tissue extraction, and mRNA expression analysis. For statistical analysis, each animal was considered the experimental unit.

### 4.13. Murine Cutaneous Wound Model

Male ICR mice, six weeks old, were obtained from ORIENT BIO (Seongnam, Republic of Korea) and acclimatized for 7 d before the experiment. The breeding environment maintained a 12 h light/dark cycle, with temperatures between 22 and 24 °C and humidity at 50 to 60%. Mice had unrestricted access to standard feed and water. Fur was removed from specific areas using an electric clipper and depilatory agent. Four 8 mm circular wounds were created in the epidermis and dermis, 2 cm from the vertebral column, using an 8 mm biopsy punch. QAABE was incorporated into a 10% sodium carboxymethyl cellulose (CMC) hydrogel at concentrations of 1, 10, and 40 mg/g. The applied concentrations (1–40 mg/g) were determined based on pilot experiments and the absence of observable adverse effects. The test material (100 μL) was applied daily for 9 d or until complete epithelialization. Digital photography documented the wounds at 0, 3, 6, and 9 d post-application, and wound sizes were measured every 3 d with a digital caliper. Four wounds were generated per animal. For statistical analysis, wound measurements from each animal were averaged and treated as one biological replicate.

### 4.14. Wound Area Histopathological Staining

After the experiment, the skin tissue around the wound was fixation with 4% paraformaldehyde for 24 h. The tissue then underwent standard histological processing, including hydration, dehydration, clearing, and infiltration, before being embedded in paraffin. It was sectioned into 4-micrometer slices and stained with H&E to assess changes in dermal tissue, such as increased epidermal thickness and inflammatory cell infiltration. Toluidine blue staining identified mast cells, while Masson’s trichrome staining evaluated dermal collagen changes. Tissue analysis was performed using optical microscopy (Leica). Each histological site scored from 0 to 3 for inflammatory cells, collagen deposition, angiogenesis, granulation tissue formation, and re-epithelialization. Histological features were semi-quantitatively evaluated on a scale ranging from 0 (none) to 4 (pronounced), according to clearly defined and reproducible criteria described by Zaini (Table 4) [35].

### 4.15. Liquid Chromatography-Tandem Mass Spectrometry (LC-MS/MS)

QAABE was dissolved to a concentration of 30 mg/mL and filtered through a 0.45 μm membrane prior to analysis. Phenolic compounds were analyzed using a Nexera X2 LC–MS/MS system (Shimadzu Corporation, Kyoto, Japan) equipped with a C18 column (Quasar, 50 × 2.1 mm, 1.7 μm, PerkinElmer, MA, USA). The mobile phase consisted of 0.1% formic acid in water (A) and 0.1% formic acid in acetonitrile (B), with a gradient from 5% B to 95% B over 10 min and maintained until 18 min. The injection volume was 5 μL with a flow rate of 0.3 mL/min. Phenolic compounds were identified and quantified using authentic reference standards (Sigma-Aldrich, St. Louis, MO, USA). Calibration curves were generated for each standard compound, and linear regression equations with correlation coefficients (R^2^) were used for quantification. Detailed calibration information is provided in the Supplementary Information (Supplementary Figure S3).

### 4.16. Statistical Analysis

Quantitative data are reported as mean ± standard deviation. For in vitro experiments, each condition was tested in triplicate, and the experiments were independently performed three to five times. For in vivo experiments, each animal was considered the experimental unit (*n* = 5 per group). Results are shown in Figure 1, Figure 2, Figure 3, Figure 4 and Figure 5. Statistical significance was assessed using one-way ANOVA and Tukey’s post hoc test with GraphPad Prism 5.0, at a significance level of *p* < 0.05.

## 5. Conclusions

To the best of our knowledge, this is the first report describing the wound-healing activity of *Quercus acuta* acorn bran extract (QAABE) in both cellular and animal models of skin wounds. However, further investigations are necessary to identify the specific pharmaceutical compounds within QAABE that are responsible for this activity. In cellular experiments, QAABE enhanced wound healing by upregulating the expression of molecular markers, including *vimentin*, *Col1a1*, *Col3a1*, *endothelin, fibronectin*, and *VEGF*, at the mRNA level. Additionally, QAABE treatment increased the protein expression of vimentin, COL1A1, endothelin, and α-SMA. Furthermore, QAABE demonstrated ROS-scavenging activity, contributing to accelerated wound healing. In a mouse model of skin injury, QAABE facilitated rapid wound recovery and exhibited anti-inflammatory properties. Further studies are warranted to validate these findings in higher-order animal models and to evaluate safety and efficacy in human clinical settings under strict ethical supervision and institutional approval. Collectively, this study provides preliminary evidence supporting the potential of QAABE as a candidate for further investigation in wound-healing research.

## Figures and Tables

**Table 1:** Histological analysis of wound healing using H&E staining and Masson’s trichrome staining with QAABE.

	Group	Vehicle (0 mg/g)	QAABE (1 mg/g)	QAABE (10 mg/g)	QAABE (40 mg/g)
Score					
Epidermis					
– Epithelialization		0	0	1	4
– Hemorrhage		3	2	1	0
– Inflammation		3	3	2	0
– Granulation tissue formation		2	2	3	4
– Neovascularization		1	2	2	3
Dermis					
– Collagen deposition		1	1	2	4

**Table 2:** Concentrations of polyphenolic compounds in QAABE.

No.	Identification	Formula	Molar Mass	mg/kg
1	Epicatechin gallate	C_22_H_18_O_10_	442.37 g/mol	4.0 ± 0.57
2	Protocatechuic acid	C_7_H_6_O_4_	154.12 g/mol	543.3 ± 104.77
3	Ellagic acid	C_14_H_6_O_8_	302.197 g/mol	22.9 ± 5.34
4	Gallic acid	C_7_H_6_O_5_	170.12 g/mol	142.6 ± 18.69
5	Isoquercitrin	C_21_H_20_O_12_	464.0955 g/mol	0.2 ± 0.03
6	Kaempferol-3-O-(2′6′-di-O-trans-p-coumaroyl)-beta-D-glucopyranoside	C_39_H_32_O_15_	740.7 g/mol	0.2 ± 0.05
7	Myricitrin	C_21_H_20_O_12_	464.37 g/mol	0.7 ± 0.12
8	Tiliroside	C_30_H_26_O_13_	594.5 g/mol	6.4 ± 1.26
9	Catechin	C_15_H_14_O_6_	290.26 g/mol	179.8 ± 28.88
10	Epicatechin	C_15_H_14_O_6_	290.27 g/mol	24.9 ± 1.46
11	Quercetin	C_15_H_10_O_7_	302.236 g/mol	23.6 ± 4.65
12	Rutin	C_27_H_30_O_16_	610.517 g/mol	3.8 ± 0.32

**Table 3:** Primer sequences used in this study.

Target Gene	Forward Primer (5′–3′)	Reverse Primer (5′–3′)
*COL1A1*	CCGTGCCCTGCCAGATC	CAGTTCTTGATTTCGTCGCAGATC
*COL3A1*	TGGAGGATGGTTGCACGAAA	AAAAGCAAACAGGGCCAACG
*VEGF-A*	ATAAGTCCTGGAGCGTTCCCT	GGCAGCGTGGTTTCTGTATC
*Vimentin*	AACTTAGGGGCGCTCTTGTC	TGAGGGCTCCTAGCGGTTTA
*Fibronectin*	ACAAGCATGTCTCTCTGCCA	TTTGCATCTTGGTTGGCTGC
*Endothelin*	CTGCCTTTTCTCCCCGTTAAA	GGACTGGGAGTGGGTTTCTC
*MMP1*	ATGCACAGCTTTCCTCCACT	GGGCCACTATTTCTCCGCTT
*TNF-α*	TAGCCCACGTCGTAGCAAAC	CTCAAAGTAGACCTGCCC
*IL-1β*	TGCCACCTTTTGACAGTGATG	AAGGTCCACGGGAAAGACAC
*IL-6*	CAACGATGATGCACTTGCAGA	TGGAAATTGGGGTAGGAAGGAC
*HPRT1*	GACCAGTCAACAGGGGACAT	GCTTGCGACCTTGACCATCT
*β-actin* *(ACTB)*	AAGGATTCCTATGTGGGCGAC	CGTACAGGGATAGCACAGCC

**Table 4:** Scoring system for histological changes in skin wound healing.

Score	0	1	2	3	4
Epithelialization	Absence of epithelial proliferation in 70% of tissue	Poor epidermal organization in 60% of tissue	Incomplete epidermal organization in 40% of tissue	Moderate epithelial proliferation in 60% of tissue	Complete epidermal remodeling in 80% of tissue
Hemorrhage	Absence of hemorrhage	1–2 per site of hemorrhage	3–4 per site of hemorrhage	5–6 per site of hemorrhage	>7 per site of hemorrhage
Granulation tissue formation	Immature and inflammatory tissue in 70% of tissue	Thin, immature, and inflammatory tissue in 60% of tissue	Moderate remodeling in 40% of tissue	Thick granulation layer in 60% of tissue	Complete tissue organization in 80% of tissue
Neovascularization	Absence of angiogenesis	1–2 vessels per site	3–4 vessels per site	5–6 vessels per site	>7 vessels per site
Collagen deposition	Absence of collagen deposition	Focal presence in fibroblasts around new capillaries	Moderate amount in repaired tissue	Dominant feature	
Inflammation	Inflammatory cell infiltration was scored as: - mild - moderate - severe				

## Data Availability

The original contributions presented in this study are included in the article/Supplementary Material. Further inquiries can be directed to the corresponding authors.

## Supplementary Materials

The following supporting information can be downloaded at: https://www.mdpi.com/article/10.3390/ph19030481/s1, Figure S1: Cell-free DCFH oxidation assay evaluating potential probe interference by QAABE.; Figure S2: LC–MS/MS MRM chromatograms of phenolic compounds identified in QAABE samples.; Figure S3: Calibration curves of phenolic reference compounds used for LC–MS/MS analysis.

## Abbreviations

The following abbreviations are used in this manuscript:

DCF-DA	2,7-dichlorofluorescein diacetate
ANOVA	Analysis of variance
CCK-8	Cell Counting Kit-8
cDNA	complementary DNA
DMSO	Dimethyl sulfoxide
DDW	Double-distilled water
DMEM	Dulbecco’s Modified Eagle’s Medium
ET-1	Endothelin-1
FBS	Fetal bovine serum
H&E	Hematoxylin and eosin
HPLC	High-performance liquid chromatography
HRP	Horseradish peroxidase
HDF	Human dermal fibroblast
H_2_O_2_	Hydrogen peroxide
Hprt1	Hypoxanthine phosphoribosyltransferase 1
ICR	Institute for Cancer Research
KFS	Korea Forest Service
LC-MS/MS	Liquid Chromatography-Tandem Mass Spectrometry
MMP1	Matrix metalloproteinase-1
NIFoS	National Institute of Forest Science
NO	Nitric oxide
QAAB	*Quercus acuta* Acorn Bran
QAABE	*Quercus acuta* acorn bran extract
qRT-PCR	Quantitative Reverse Transcription Polymerase Chain Reaction
ROS	Reactive oxygen species
TGF	Transforming growth factor
TNF-α	Tumor necrosis factor-alpha
VEGF	Vascular endothelial growth factor
α-SMA	α-smooth muscle actin

## References

[1] Lin T.K., Zhong L., Santiago J.L. (2017). Anti-Inflammatory and Skin Barrier Repair Effects of Topical Application of Some Plant Oils. Int. J. Mol. Sci..

[2] Ibrahim N., Wong S.K., Mohamed I.N., Mohamed N., Chin K.Y., Ima-Nirwana S., Shuid A.N. (2018). Wound Healing Properties of Selected Natural Products. Int. J. Environ. Res. Public Health.

[3] Wallace H.A., Basehore B.M., Zito P.M. (2025). Wound Healing Phases. StatPearls.

[4] Karppinen S.M., Heljasvaara R., Gullberg D., Tasanen K., Pihlajaniemi T. (2019). Toward understanding scarless skin wound healing and pathological scarring. F1000Research.

[5] Singer A.J. (2022). Healing Mechanisms in Cutaneous Wounds: Tipping the Balance. Tissue Eng. Part B Rev..

[6] Scialò F., Fernández-Ayala D.J., Sanz A. (2017). Role of Mitochondrial Reverse Electron Transport in ROS Signaling: Potential Roles in Health and Disease. Front. Physiol..

[7] Rodriguez P.G., Felix F.N., Woodley D.T., Shim E.K. (2008). The Role of Oxygen in Wound Healing: A Review of the Literature. Dermatol. Surg..

[8] Cano Sanchez M., Lancel S., Boulanger E., Neviere R. (2018). Targeting Oxidative Stress and Mitochondrial Dysfunction in the Treatment of Impaired Wound Healing: A Systematic Review. Antioxidants.

[9] Ponugoti B., Xu F., Zhang C., Tian C., Pacios S., Graves D.T. (2013). FOXO1 promotes wound healing through the up-regulation of TGF-β1 and prevention of oxidative stress. J. Cell Biol..

[10] Dunnill C., Patton T., Brennan J., Barrett J., Dryden M., Cooke J., Leaper D., Georgopoulos N.T. (2017). Reactive oxygen species (ROS) and wound healing: The functional role of ROS and emerging ROS-modulating technologies for augmentation of the healing process. Int. Wound J..

[11] Comino-Sanz I.M., Lopez-Franco M.D., Castro B., Pancorbo-Hidalgo P.L. (2021). The Role of Antioxidants on Wound Healing: A Review of the Current Evidence. J. Clin. Med..

[12] Woodruff L.D., Bounkeo J.M., Brannon W.M., Dawes K.S., Barham C.D., Waddell D.L., Enwemeka C.S. (2004). The efficacy of laser therapy in wound repair: A meta-analysis of the literature. Photomed. Laser Surg..

[13] Chester D., Marrow E.A., Daniele M.A., Brown A.C., Narayan R. (2019). Wound Healing and the Host Response in Regenerative Engineering. Encyclopedia of Biomedical Engineering.

[14] Yoon I.S., Park D.H., Bae M.S., Oh D.S., Kwon N.H., Kim J.E., Choi C.Y., Cho S.S. (2017). In Vitro and In Vivo Studies on *Quercus acuta* Thunb. (Fagaceae) Extract: Active Constituents, Serum Uric Acid Suppression, and Xanthine Oxidase Inhibitory Activity. Evid. Based Complement. Alternat. Med..

[15] Kim M.H., Park D.H., Bae M.S., Song S.H., Seo H.J., Han D.G., Oh D.S., Jung S.T., Cho Y.C., Park K.M. (2018). Analysis of the Active Constituents and Evaluation of the Biological Effects of *Quercus acuta* Thunb. (Fagaceae) Extracts. Molecules.

[16] Cho S.H., Kim K.J., Park C.W., Sun B.Y., Chung M.G., Pak J.H. (2008). Cuticle micromorphology of Leaves of *Quercus* L. (Fagaceae) and its taxonomic implications. Korean J. Plant Taxon..

[17] Denk T., Grimm G.W., Manos P.S., Deng M., Hipp A.L., Gil-Pelegrín E., Peguero-Pina J.J., Sancho-Knapik D. (2017). An Updated Infrageneric Classification of the Oaks: Review of Previous Taxonomic Schemes and Synthesis of Evolutionary Patterns. Oaks Physiological Ecology. Exploring the Functional Diversity of Genus *Quercus* L..

[18] Uyar A., Jhangir G.M., Keleş Ö.F., Yener Z. (2023). The effects of *Quercus* (Oak) acorn on cutaneous wound healing in rats. Int. J. Plant Based Pharm..

[19] Kim S.H., Shin H.L., Son T.H., Lim S.A., Kim D., Yoon J.H., Choi H., Kim H.G., Choi S.W. (2024). *Quercus glauca* Acorn Seed Coat Extract Promotes Wound Re-Epithelialization by Facilitating Fibroblast Migration and Inhibiting Dermal Inflammation. Biology.

[20] Cheng F., Shen Y., Mohanasundaram P., Lindstrom M., Ivaska J., Ny T., Eriksson J.E. (2016). Vimentin coordinates fibroblast proliferation and keratinocyte differentiation in wound healing via TGF-beta-Slug signaling. Proc. Natl. Acad. Sci. USA.

[21] Hochstein A.O., Bhatia A. (2014). Collagen: Its role in wound healing. Wound Manag..

[22] Barton M., Yanagisawa M. (2019). Endothelin: 30 Years From Discovery to Therapy. Hypertension.

[23] Shinde A.V., Humeres C., Frangogiannis N.G. (2017). The role of α-smooth muscle actin in fibroblast-mediated matrix contraction and remodeling. Biochim. Biophys. Acta (BBA)—Mol. Basis Dis..

[24] Wang G., Yang F., Zhou W., Xiao N., Luo M., Tang Z. (2023). The initiation of oxidative stress and therapeutic strategies in wound healing. Biomed. Pharmacother..

[25] Lenselink E.A. (2015). Role of fibronectin in normal wound healing. Int. Wound J..

[26] Bao P., Kodra A., Tomic-Canic M., Golinko M.S., Ehrlich H.P., Brem H. (2009). The role of vascular endothelial growth factor in wound healing. J. Surg. Res..

[27] Guo S., Dipietro L.A. (2010). Factors affecting wound healing. J. Dent. Res..

[28] Dunn L., Prosser H.C., Tan J.T., Vanags L.Z., Ng M.K., Bursill C.A. (2013). Murine model of wound healing. J. Vis. Exp..

[29] Said A., Wahid F., Bashir K., Rasheed H.M., Khan T., Hussain Z., Siraj S. (2019). Sauromatum guttatum extract promotes wound healing and tissue regeneration in a burn mouse model via up-regulation of growth factors. Pharm. Biol..

[30] Wu J., Landen N.X. (2020). Investigation of Skin Wound Healing Using a Mouse Model. Methods Mol. Biol..

[31] Yadav E., Singh D., Yadav P., Verma A. (2017). Attenuation of dermal wounds via downregulating oxidative stress and inflammatory markers by protocatechuic acid rich n-butanol fraction of Trianthema portulacastrum Linn. in wistar albino rats. Biomed. Pharmacother..

[32] Baek S., Park H., Kim M., Lee D. (2020). Preparation of PCL/(+)-catechin/gelatin film for wound healing using air-jet spinning. Appl. Surf. Sci..

[33] Rozen S., Skaletsky H. (2000). Primer3 on the WWW for general users and for biologist programmers. Methods Mol. Biol..

[34] Livak K.J., Schmittgen T.D. (2001). Analysis of relative gene expression data using real-time quantitative PCR and the 2^−ΔΔCT^ Method. Methods.

[35] Zaini A.A., Khaza’ai H., Ali R.M., Abdul Mutalib M.S., Baharuddin A.A. (2016). Topical Treatment of Tocotrienol-Rich Fraction ( TRF ) on Deep Partial-Thickness Burn Wounds in Rats. J. Dermatol. Clin. Res..

