# Supplementary material for: Quercus acuta Acorn Bran Extract Enhances Wound Healing by Promoting Human Dermal Fibroblast Migration and Antioxidant Activity
Source: Pharmaceuticals (Basel). 2026 Mar 15;19(3):481. doi: 10.3390/ph19030481 (PMC13029716; doi:10.3390/ph19030481)
Supplement: Supplementary file 1 [file pharmaceuticals-19-00481-s001.zip › pharmaceuticals-4146000-supplementary.pdf]

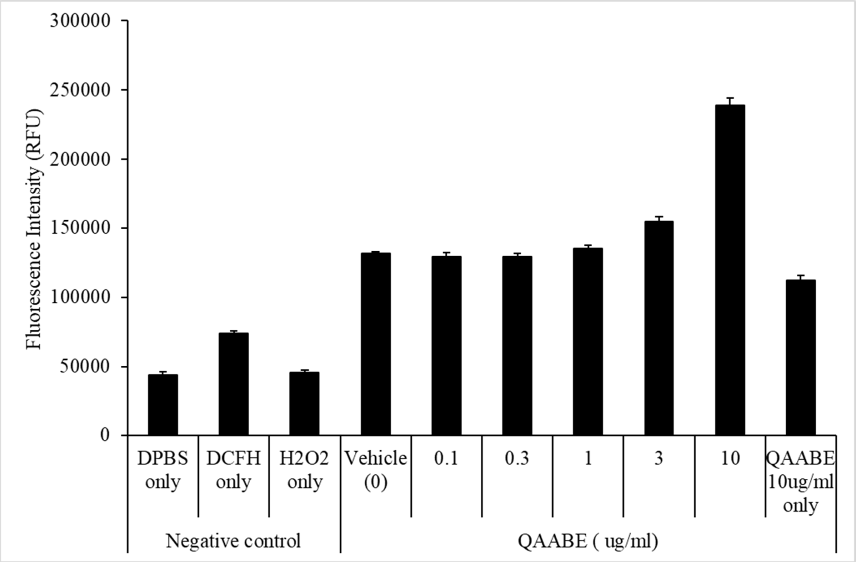

Supplementary Figure S1. Cell-free DCFH oxidation assay evaluating potential probe interference by QAABE. DCFH oxidation was induced by H_2O_2 in the absence of cells, and fluorescence intensity was measured in the presence of increasing concentrations of QAABE. Vehicle represents the DCFH + H_2O_2 control containing DMSO. QAABE did not suppress DCF fluorescence; instead, fluorescence intensity increased with increasing QAABE concentrations. QAABE-only wells (DPBS + QAABE) were included to evaluate extract-derived background fluorescence. Although QAABE alone produced a modest fluorescence signal compared with DPBS, this signal remained lower than the fluorescence observed in all DCFH + H_2O_2–containing conditions. These results indicate that QAABE does not act as a fluorescence quencher of the DCF probe.

Commented [M1]: We remove the first page of the supplementary materials according to our guidelines. Please confirm.

Commented [TS2R1]: I confirm

https://doi.org/10.3390/xxxxx

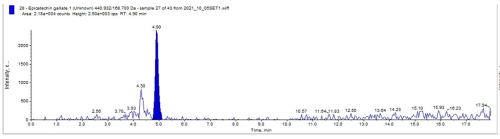

Epicatechin gallate

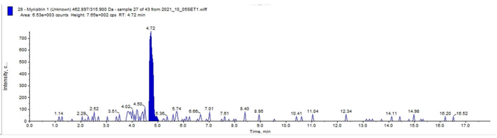

Myricitrin

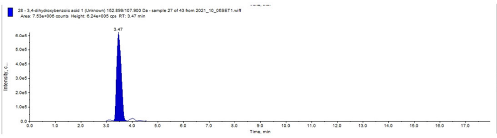

Protocatechuic acid

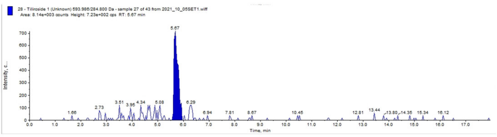

Tiliroside

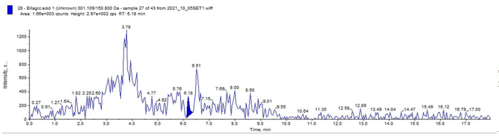

Ellagic acid

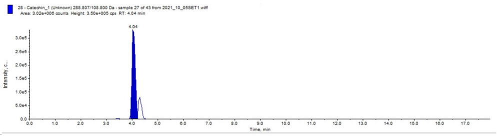

Catechin

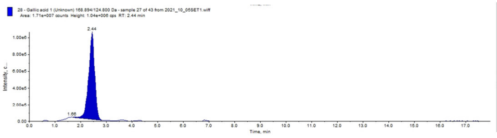

Gallic acid

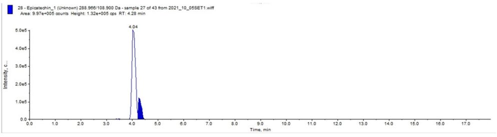

Epicatechin

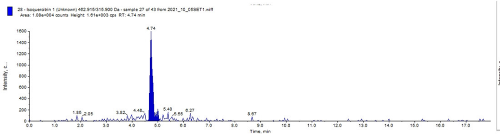

Isoquercitrin

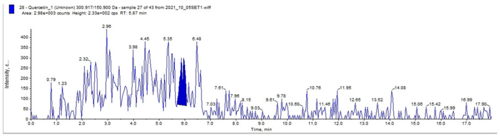

Quercetin

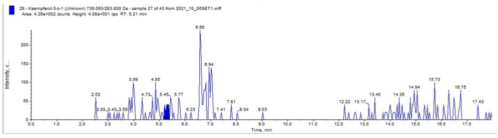

Kaempferol-3-O-(2'6'-di-O-trans-p-coumaroyl)-beta-D-glucopyranoside

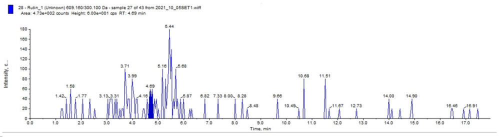

Rutin

Supplementary Figure S2. LC–MS/MS MRM chromatograms of phenolic compounds identified in QAABE samples. Each peak represents the characteristic precursor-to-product ion transition of the respective compound.

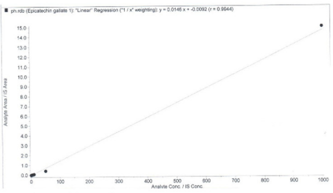

Epicatechin gallate

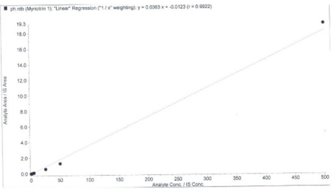

Myricitrin

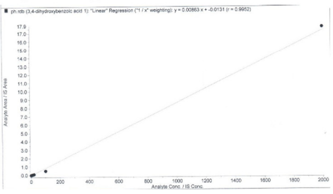

Protocatechuic acid

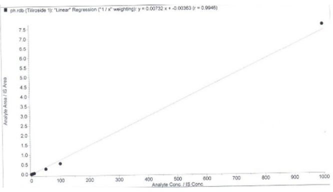

Tiliroside

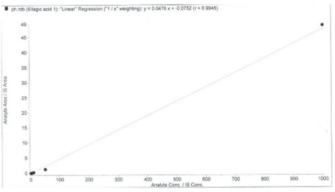

Ellagic acid

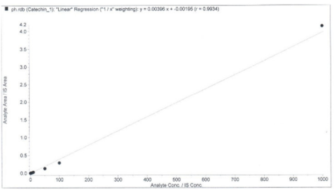

Catechin

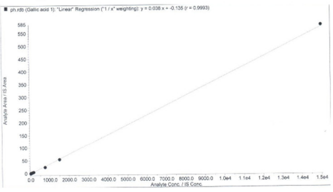

Gallic acid

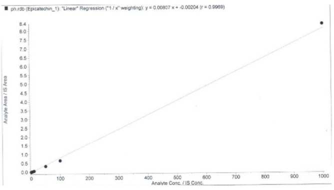

Epicatechin

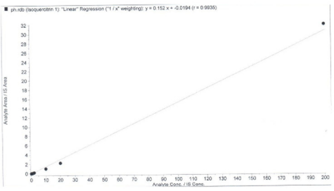

Isoquercitrin

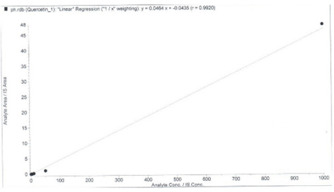

Quercetin

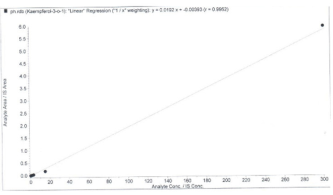

Kaempferol-3-O-(2'6'-di-O-trans-p-coumaroyl)-beta-D-glucopyranoside

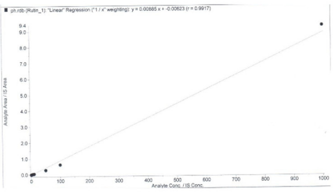

Rutin

Supplementary Figure S3. Calibration curves of phenolic reference compounds used for LC–MS/MS analysis. Calibration curves were generated using authentic reference standards analyzed under the same LC–MS/MS conditions as the QAABE samples. Linear regression equations and correlation coefficients (R^2) were calculated for each compound and used for the quantification of phenolic compounds detected in QAABE.
